# ﻿A review of the genus *Vitrea* Fitzinger, 1833 (Gastropoda, Eupulmonata, Pristilomatidae) in Serbia: diversity, distribution and the description of a new species

**DOI:** 10.3897/zookeys.1200.120633

**Published:** 2024-05-09

**Authors:** Vukašin Gojšina, Nikola Vesović, Srećko Ćurčić, Tamara Karan-Žnidaršič, Biljana Mitrović, Ivaylo Dedov

**Affiliations:** 1 University of Belgrade - Faculty of Biology, Studentski Trg 16, 11000 Belgrade, Serbia University of Belgrade - Faculty of Biology Belgrade Serbia; 2 The Museum of Natural History, Njegoševa 51, 11000 Belgrade, Serbia The Museum of Natural History Belgrade Serbia; 3 Institute of Biodiversity and Ecosystem Research, Bulgarian Academy of Sciences, 2 Gagarin Street, 1113 Sofia, Bulgaria Institute of Biodiversity and Ecosystem Research, Bulgarian Academy of Sciences Sofia Bulgaria

**Keywords:** faunistics, Mt. Devica, pit, taxonomy, terrestrial snails

## Abstract

In this paper, the genus *Vitrea* Fitzinger, 1833 in Serbia is reviewed. All previous literature data on this genus from Serbia are summarised and used to discuss its distribution in the country and create distribution maps, supplemented by new material collected by the authors. All Serbian species are figured. For each species, a brief description of the examined specimens, data on previous findings in Serbia, the material (including types) that were analysed, the distribution and habitats in Serbia they inhabit, as well as remarks on specific species are given. A new species, *Vitreavirgo* Gojšina & Dedov, **sp. nov.**, is described from a pit on Mt. Devica in eastern Serbia. *Vitreapygmaea* (O. Boettger, 1880) is reported for the first time for the territory of Serbia. As some *Vitrea* species have a narrow geographical range and prefer certain habitats, they are particularly vulnerable to habitat changes, which is also discussed in the paper. An identification key for all hitherto known Serbian species is given.

## ﻿Introduction

*Vitrea* Fitzinger, 1833 is a genus of tiny terrestrial pulmonate gastropods with a shell width (SW) < 6 mm, usually with an unpigmented body and a translucent shell (Welter-Schultes 2012). The genus is widespread in Europe and extends eastwards all the way to Iran (Riedel 1966; Sysoev and Schileyko 2009; Welter-Schultes 2012). The southernmost known localities are situated in North Africa (Pintér 1969; Riedel 1976). Although there are several relatively widespread species, most species are known from limited geographical areas (Welter-Schultes 2012; Páll-Gergely and Asami 2015). The genus is the richest in species within the family Pristilomatidae, with a total of 78 extant species (MolluscaBase 2024), ~ 40 of which inhabit the Balkans (Pintér 1972; Welter-Schultes 2012). Several species are described and known exclusively from caves (Wagner 1914; Riedel and Velkovrh 1976; Pintér 1983), while several others occasionally inhabit caves and are usually restricted to limestone habitats (Pintér 1972; Riedel 1984).

The three most species-rich European pristilomatid genera (*Gyralina* Andreae, 1902, *Lindbergia* A. Riedel, 1959, and *Vitrea*) are relatively well-separated from each other conchologically. The most important conchological difference between *Lindbergia* and *Vitrea* lies in the size of the shell (the shell of the former is larger). *Gyralina* has a peculiar shell surface in the form of strong spiral striae and a *Nautilus*-like protruded apertural margin (Welter-Schultes 2012). There are also clear differences between them in their genital anatomy. In contrast to *Gyralina* and *Lindbergia*, *Vitrea* has no epiphallus and its seminal receptacle is reduced or absent. These two structures are well-developed in *Gyralina* and *Lindbergia*. The genus *Spinophallus* A. Riedel, 1962 also has a well-developed seminal receptacle, but is additionally characterised by the presence of conical papillae inside the penis (Schileyko 2003).

*Vitrea* is the only pristilomatid genus in Serbia and is represented by a total of eight species in the country: *V.contracta* (Westerlund, 1871), *V.crystallina* (O. F. Müller, 1774), *V.diaphana* (S. Studer, 1820), *V.illyrica* (A. J. Wagner, 1907), *V.kiliasi* L. Pintér, 1972, *V.kutschigi* (Walderdorff, 1864), *V.sturanyi* (A. J. Wagner, 1907), and *V.subrimata* (Reinhardt, 1871) (Karaman 2007). The first to provide comprehensive data on the distribution of *Vitrea* species in Serbia was Pavlović (1912). In his work, he listed a total of five *Vitrea* species in the country, all of which he assigned to the genus *Crystallus* R. T. Lowe, 1855, a synonym of *Vitrea*. His data were summarised by Tomić (1959). Jaeckel et al. (1957) provided data on species already recorded in Serbia, with no new faunistic records provided. Pintér (1972) revised the genus *Vitrea* from the Balkans and reported new sampling sites from Serbia. Karaman provided further data on the distribution of *Vitrea* species in Serbia in several faunistic papers (Jovanović 1985, 1993, 1996; Karaman 2007, 2012).

The aims of this paper are to: (i) list all species of the genus *Vitrea* in Serbia, (ii) discuss their distribution and occurrence in the country, (iii) describe a new species, *V.virgo* Gojšina & Dedov, sp. nov., and (iv) present an identification key for all known *Vitrea* species in Serbia.

## ﻿Materials and methods

Most of the snails were collected by the authors (VG, NV, SĆ) from 2021 to 2023, with special attention paid to numerous limestone habitats in eastern Serbia and several of them in western Serbia (altogether 30 sampling sites). This sampling included several localities already visited by Academician Petar S. Pavlović, as well as hitherto unknown sites. The northern part of the country (the Autonomous Province of Vojvodina) was not thoroughly sampled as this region is mostly covered by agricultural fields and almost completely devoid of limestone. Snails were collected manually or were sorted from soil samples under a stereomicroscope. Occasionally, soil was sieved *in situ* and snails were collected immediately. Living animals were preserved in 70% ethanol and labelled accordingly. The shells and genitalia (stored in 70% ethanol) were photographed using a Zeiss SteREO Discovery.V12 stereomicroscope equipped with a Leica Flexacam C3 camera and a Nikon SMZ800N stereomicroscope equipped with a Nikon DS-Fi2 camera. A Nikon DS-L3 control unit was used to set scale bars. Shell microsculpture of the newly described species was imaged using a Jeol JSM-6390LV scanning electron microscope. The sample was gold-coated under 30 mA for 100 sec using a Bal-Tec SCD 005 sputter coater. Type specimens are deposited in The Museum of Natural History (Belgrade, Serbia) (**NHMBEO**), Institute of Biodiversity and Ecosystem Research (Sofia, Bulgaria) (**IBER**), and Institute of Zoology, University of Belgrade - Faculty of Biology (Belgrade, Serbia) (**IZOO**) collections. The type specimens of *V.illyrica*, *V.kutschigi*, and *V.sturanyi* from the Senckenberg Forschungsinstitut und Naturmuseum (Frankfurt am Main, Germany) (**SMF**) collection were processed and photographed. Part of the *Vitrea* collection of Petar S. Pavlović, deposited in the NHMBEO collection, was also examined (see under the Material examined section for each species). The paratypes of *Vitreasiveci* Riedel & Velkovrh, 1976 and non-type specimen of *V.kiliasi* L. Pintér, 1972, which are deposited in the Museum and Institute of Zoology of the Polish Academy of Sciences (Warsaw, Poland) (**MIZ**) collection, were also used for comparison with the new species. The photos of these two species were taken with a Keyence VHX-7000 digital microscope. Distribution maps were created using data from published literature sources (Möllendorff 1873; Pavlović 1912; Tomić 1959; Pintér 1972; Jovanović 1985, 1993, 1996; Sólymos et al. 2004; Karaman 2012) and newly obtained data. Nomenclature follows MolluscaBase (2024).

Abbreviations used in the text are as follows:

**AH** aperture height

**AW** aperture width

**IBER**Institute of Biodiversity and Ecosystem Research, Bulgarian Academy of Sciences, Sofia, Bulgaria

**IZOO**Institute of Zoology, University of Belgrade - Faculty of Biology, Belgrade, Serbia

**MIZ**Museum and Institute of Zoology of the Polish Academy of Sciences, Warsaw, Poland

**NHMBEO** The Museum of Natural History, Belgrade, Serbia

**SH** shell height

**SMF** Senckenberg Forschungsinstitut und Naturmuseum (Frankfurt am Main, Germany)

**SW** shell width

**UW** umbilicus width

## ﻿Results

### ﻿Taxonomic account

**Class Gastropoda Cuvier, 1795**

**Superorder Eupulmonata Haszprunar & Huber, 1990**

**Order Stylommatophora A. Schmidt, 1855**

**Superfamily Gastrodontoidea Tryon, 1866**

**Family Pristilomatidae Cockerell, 1891**

#### ﻿Genus Vitrea

Taxon classificationAnimaliaStylommatophoraPristilomatidae

Fitzinger, 1833

4E6FC399-E824-5938-8F35-FCAB5B6FEC96

##### Type species.

Glischrus (Helix) diaphana S. Studer, 1802, by monotypy.

#### ﻿ Vitrea contracta

Taxon classificationAnimaliaStylommatophoraPristilomatidae

(Westerlund, 1871)

B10BB9D7-705F-5964-AE97-B2638A9E4E94

Figs 1 , 15

Crystallus contractus subcontractus — Pavlović 1912: 26.
Crystollus cintractus [sic] — Tomić 1959: 13.
Vitrea contracta — Pintér 1972: 274; Jovanović 1985: 42; Sólymos et al. 2004: 152; Karaman 2012: 24.
Vitrea contracta contracta — Karaman 2007: 141.
Vitrea contracte [sic] — Jovanović 1996: 219.

##### Sites in Serbia known from the literature.

After Pavlović (1912) and Tomić (1959): Topčider, city of Belgrade; Velika Tisnica Gorge, near the town of Žagubica; Metino Brdo hill, near the city of Kragujevac; Sveti Stefan (Lipovac) Monastery, near the town of Aleksinac; Prekonoško Vrelo, village of Prekonoga, near the town of Svrljig; village of Crnoljevica, near the town of Svrljig; Sirinjava Duvka, near the village of Periš, Svrljiške Planine Mts.; Jevik hill, near the town of Knjaževac; Mt. Stol, near the city of Bor; after Pintér (1972): town of Raška; near the town of Sokobanja; Sveta Petka Monastery, near the city of Niš; after Jovanović (1985): Mt. Avala, near the city of Belgrade; after Jovanović (1996): Mt. Stol, near the city of Bor; after Sólymos et al. (2004) and Karaman (2012): near the Dobri Potok stream, Mt. Fruška Gora.

**Figure 1.**
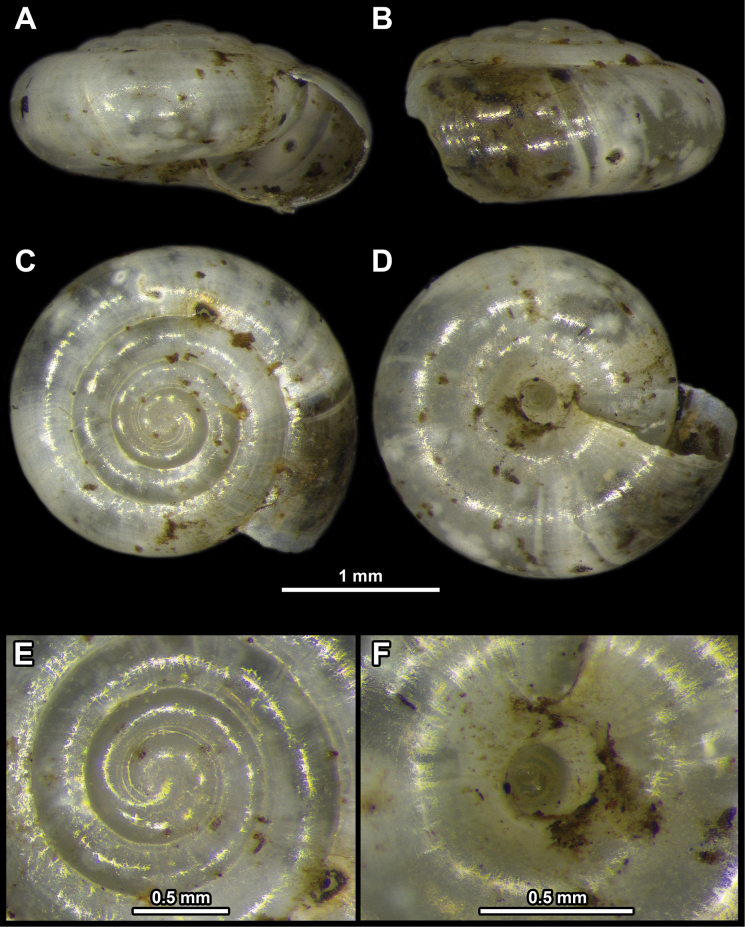
*Vitreacontracta* from the surroundings of the city of Pirot **A** apertural view **B** lateral view **C** apical view **D** umbilical view **E** enlarged view of the protoconch **F** enlarged view of the umbilicus.

##### Material examined.

**Serbia** • Near the town of Svrljig, village of Crnoljevica, leg. P. Pavlović, one specimen (NHMBEO442); Mt. Stol, 26 Sep. 1907, three specimens (NHMBEO445); surroundings of the city of Pirot, a hill above Kitka rock quarry, among rocks, leg. V. Gojšina, M. Vujić & N. Vesović, 28 Apr. 2023, one specimen (43°11'19.65"N, 22°38'47.14"E); Stara Planina Mts., Babin Zub peak, leg. V. Gojšina, M. Vujić & N. Vesović, 07 May 2023, 12 specimens (43°22'25.79"N, 22°36'46.30"E); Felješana Strict Nature Reserve, near the settlement of Debeli Lug, leg. V. Gojšina, M. Vujić & N. Vesović, 03 Jun. 2023, one specimen (44°20'36.48"N, 21°53'20.57"E); Đerdap National Park, village of Dobra, leg. V. Gojšina, M. Vujić & N. Vesović, 05 May 2023, two specimens (44°38'27.53"N, 21°54'29.38"E).

##### Description of specimens from Serbia.

Shell very small, consisting of 4–5 whorls, colourless, translucent, SW usually ~ 2 mm, but ≤ 3 mm. Shell surface smooth. Last whorl ~ 1.5× as wide as penultimate whorl. Umbilicus moderately broad and widening near last whorl, revealing almost all whorls.

##### Distribution and habitats in Serbia.

Mostly found in dry, karstic habitats among rocks. Not frequently found in Serbia, probably overlooked due to its small size. Most records came from eastern Serbia (Fig. 15), otherwise with scarce findings. This species is more widespread in the country, which should be proven by further research.

#### ﻿ Vitrea crystallina

Taxon classificationAnimaliaStylommatophoraPristilomatidae

(O. F. Müller, 1774)

3A7513A1-DE0F-50FE-AE43-51A172425B82

Figs 2 , 15

Hyalina crystallina — Möllendorff 1873: 131.
Vitrea crystallina — Hesse 1929: 235; Jaeckel et al. 1957: 156; Karaman 2007: 141.

##### Sites in Serbia known from the literature.

After Möllendorff (1873): Mt. Javor; Rača Monastery, Mt. Tara.

##### Material examined.

**Serbia** • Village of Deliblato, next to the Kraljevac Lake, leg. V. Gojšina, 11 Oct. 2020, one specimen (44°50'58.44"N, 21°01'17.75"E); city of Belgrade, Kalemegdan fortress, leg. M. Vujić, 28 Dec. 2022, one specimen (44°49'19.23"N, 20°27'02.79"E); town of Sokobanja, village of Resnik, near a spring, leg. V. Gojšina & M. Vujić, 07 Nov. 2023, seven specimens (43°37'57.79"N, 21°48'55.28"E).

**Figure 2.**
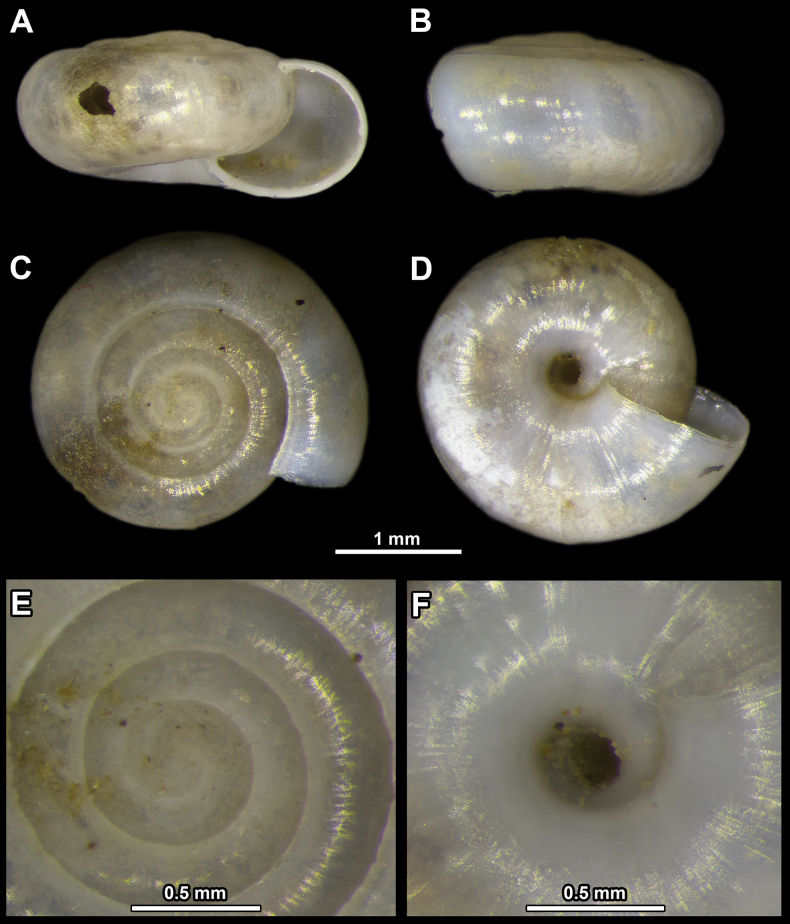
*Vitreacrystallina* from the village of Deliblato **A** apertural view **B** lateral view **C** apical view **D** umbilical view **E** enlarged view of the protoconch **F** enlarged view of the umbilicus.

##### Description of specimens from Serbia.

Shell up to 3–4 mm wide, colourless, transparent, consisting of 4–5 whorls, which are not densely coiled. Last whorl twice as wide as penultimate whorl. Periphery rounded. Umbilicus open and moderately broad, widening at last whorl. Only penultimate whorl clearly visible through umbilicus.

##### Distribution and habitats in Serbia.

Poorly known from Serbia due to a lack of sampling. It is known from western Serbia, the surroundings of the city of Belgrade and Deliblato Sands (Fig. 15).

#### ﻿ Vitrea diaphana

Taxon classificationAnimaliaStylommatophoraPristilomatidae

(S. Studer, 1820)

410B8D0B-CB28-5B60-9556-5B41C3F3BB77

Figs 3 , 16

Crgstallus diaphanus [sic] — Pavlović 1912: 24–25.
Crystllus diaphanus [sic] — Tomić 1959: 12.
Vitrea diaphana — Jaeckel et al. 1957: 156; Jovanović 1985: 42; Jovanović 1993: 241–242; Jovanović 1996: 219; Karaman 2007: 141; Karaman 2012: 24.
Vitrea diaphana diaphana — Pintér 1972: 214; Sólymos et al. 2004: 152.

##### Sites in Serbia known from the literature.

After Pavlović (1912) and Tomić (1959): Topčider, city of Belgrade; Mt. Beljanica; Velika Tisnica Gorge, near the town of Žagubica; Mt. Vujan, near the town of Gornji Milanovac; Mt. Vidlič, near the city of Pirot; near the Sveta Petka Monastery, Grza Gorge, near the town of Paraćin; Jelašnica Gorge, near the city of Niš; Mt. Javor; next to the Dubočica river, near the town of Raška; Radmanov Kamen, Mt. Kopaonik; around the Pogana Peć Cave, near the village of Krepoljin; Koprivštički Krst, near the city of Pirot; village of Lunjevica, near the town of Gornji Milanovac; Rajkovo, near the town of Majdanpek; Mali Štrbac peak, Mt. Miroč; Mt. Ovčar; near the village of Periš, Svrljiške Planine Mts.; Mt. Rtanj; Stenka peak, near the town of Paraćin; Mt. Suva Planina; Sićevo Gorge; Milenkova Stena, Svrljiške Planine Mts.; Pleš peak, Svrljiške Planine Mts.; village of Niševac, near the town of Svrljig; Mt. Stol, near the city of Bor; Glogovački Vrh peak, Mt. Tupižnica; village of Tumba, near the city of Vranje; Crnica Gorge, near the town of Paraćin; Mt. Crni Vrh, near the city of Jagodina; village of Crnoljevica, near the town of Svrljig; after Pintér (1972): Veta Monastery, Mt. Suva Planina; town of Sokobanja; after Jovanović (1985): Mt. Avala, near the city of Belgrade; after Jovanović (1993): Mt. Veliki Krš, near the city of Bor; Mikuljska Reka river canyon, village of Zlot, near the city of Bor; after Jovanović (1996): Mt. Stol, near the city of Bor; after Sólymos et al. (2004) and Karaman (2012): near the Dobri Potok stream, Mt. Fruška Gora.

**Figure 3.**
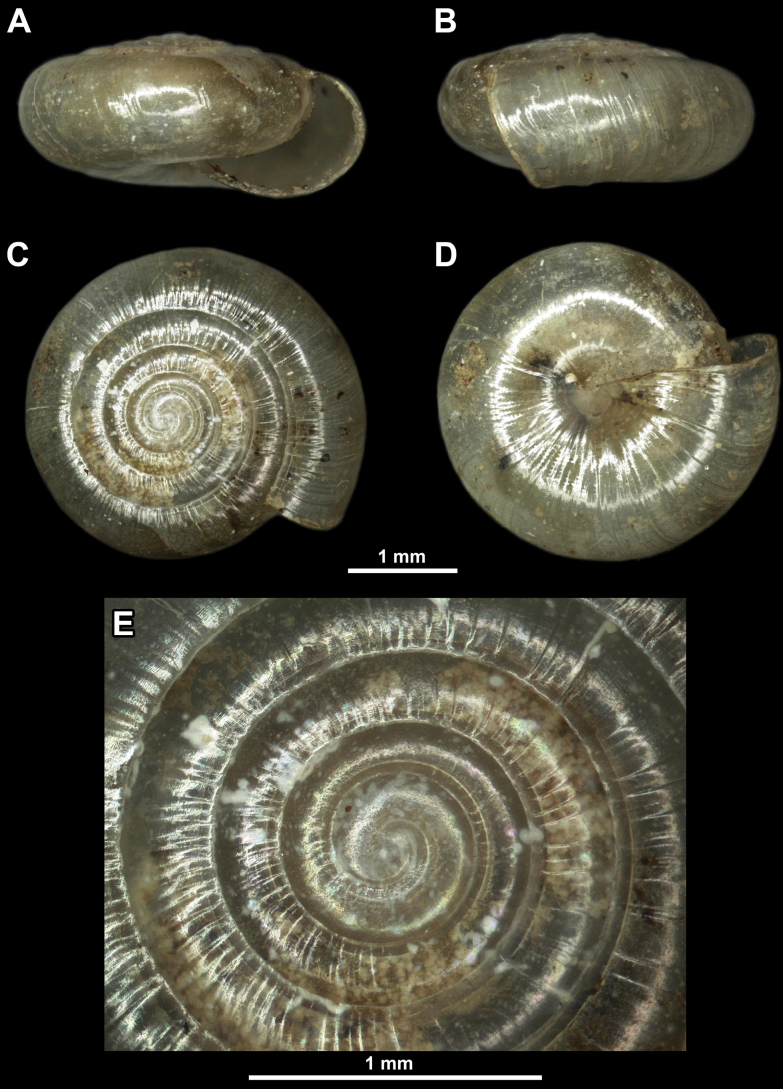
*Vitreadiaphana* from the surroundings of the city of Pirot **A** apertural view **B** lateral view **C** apical view **D** umbilical view **E** enlarged view of the protoconch.

##### Material examined.

**Serbia** • Sićevo Gorge, leg. P. Pavlović, 30 Sep. 1906, one specimen (NHMBEO371); Vlasina Landscape of Outstanding Features, Mt. Vardenik, leg. V. Gojšina & M. Vujić, 04 Jun. 2022, one specimen (42°37'53.80"N, 22°16'51.00"E); Vlasina Landscape of Outstanding Features, Mt. Čemernik, next to the Cvetkova Reka river, leg. V. Gojšina, 02 June 2022, one specimen (42°44'41.12"N, 22°18'50.59"E); Vlasina Landscape of Outstanding Features, next to the Vučja Reka river, leg. V. Gojšina, 03 June 2022, one specimen (42°45'12.69"N, 22°23'51.70"E); village of Krivelj, near Mt. Veliki Krš, leg. V. Gojšina, 19 Jun. 2022, one specimen (44°10'07.00"N, 22°06'24.25″E); town of Bela Palanka, settlement of Čiflik, near the Sinjac Monastery, leg. V. Gojšina, 05 Aug. 2022, one specimen (43°13'03.62"N, 22°24'54.45"E); Crni Timok Gorge, village of Krivi Vir, leg. M. Šćiban, 03 May 2012, three specimens; Mt. Golija, village of Devići, leg. V. Gojšina, 25 Jul. 2022, one specimen (43°25'44.6"N, 20°22'38.6"E); Jelašnica Gorge, near the city of Niš, on limestone rocks, leg. V. Gojšina, 28 May 2022, two specimens (43°16'45.82"N, 22°03'49.59"E); Stara Planina Mts., village of Temska, near the Bukovički Do waterfall, sieved from leaf litter in a limestone rock crevice, leg. V. Gojšina, 30 May 2022, two specimens (43°16'41.12"N, 22°34'10.25"E); Mt. Suva Planina, Bojanine Vode, sieved from leaf litter, leg. V. Gojšina, 31 May 2022, five specimens (43°13'13.56"N, 22°06'52.66"E); Stara Planina Mts., near the Bigar waterfall, leg. V. Gojšina, 05 Aug. 2022, three specimens (43°21'16.13"N, 22°26'33.02"E); city of Pirot, near the village of Dobri Do, Kitka rock quarry, leg. V. Gojšina, M. Vujić & N. Vesović, 28 Apr. 2023, one specimen (43°11'19.58"N, 22°38'47.31"E); Stara Planina Mts., Babin Zub peak, leg. V. Gojšina, M. Vujić & N. Vesović, 07 May 2023, two specimens (43°22'25.79"N, 22°36'46.30"E); near the town of Vrnjačka Banja, an oak forest, leg. V. Gojšina, 24 Mar. 2023, five specimens (43°35'15.76"N, 20°54'23.98"E); outskirts of the town of Vrnjačka Banja, near a small brook, leg. V. Gojšina, 24 Mar. 2023, one specimen (43°35'19"N, 20°54'25"E).

##### Description of specimens from Serbia.

SW ranging from 3.5 up to even 5 mm. Shell surface smooth, with relatively strong radial growth lines. Shell transparent and flat, consisting of 5–6 relatively densely coiled whorls separated by shallow suture. Periphery rounded. Last whorl ~ 2× as wide as penultimate whorl. Umbilicus entirely closed.

##### Distribution and habitats in Serbia.

Together with *V.subrimata*, this is the most common and widespread *Vitrea* species in Serbia (Fig. 16). Most frequently found in areas rich in limestone.

##### Remarks.

Particularly large specimens (SW nearly 5 mm) were found at Bojanine Vode site on Mt. Suva Planina. Pavlović (1912) mentioned that he found several specimens in different locations (Jelašnica Gorge, Sirinjava Duvka, Ulanac peak on Svrljiške Planine Mts.) that represent a form of *V.diaphana* with a very narrow umbilicus (even narrower than in *V.subrimata*) or possibly an undescribed species. In Jelašnica Gorge, we found both *V.diaphana* and *V.subrimata*, which makes it more likely that it is indeed *V.diaphana* with a not yet fully closed umbilicus. We have not found any specimens that fit Pavlović’s description. The specimens he collected from the village of Sićevo (NHMBEO371) are not properly cleaned and could represent *V.subrimata*. The samples of *V.diaphana* collected by Pavlović (NHMBEO364 and NHMBEO365) are missing from the NHMBEO collection.

#### ﻿ Vitrea illyrica

Taxon classificationAnimaliaStylommatophoraPristilomatidae

(A. J. Wagner, 1907)

6F944EF1-C321-59B2-9855-92B7D83217E3

Figs 4 , 15

Crystallus illyricus — Pavlović 1912: 26–27; Tomić 1959: 13.
Vitrea illyrica — Jaeckel et al. 1957: 156; Karaman 2007: 141.

##### Sites in Serbia known from the literature.

After Pavlović (1912) and Tomić (1959): Derventa river canyon, Mt. Tara; Drundebo, Mt. Tara; Mt. Javor; Krstača, near the Rača Monastery, Mt. Tara; near the Perućac Lake, Mt. Tara; Mt. Povlen.

**Figure 4.**
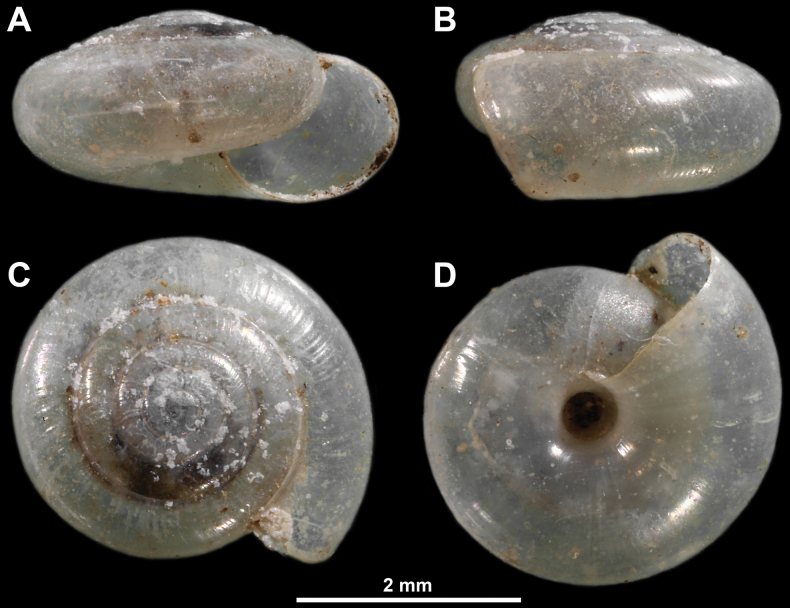
Paralectotype of *Vitreaillyrica* from Scutari, Albania (SMF171013) **A** apertural view **B** lateral view **C** apical view **D** umbilical view.

##### Types examined.

Scutari, Albania, three paralectotypes (SMF171013).

##### Other material examined.

**Serbia** • Mt. Javor, leg. P. Pavlović, 1908, nine specimens (NHMBEO452); Mt. Tara, Drundebo, leg. P. Pavlović, 07–12 Aug. 1909, three specimens (NHMBEO450) (see under the Remarks for *V.kutschigi*); Mt. Povlen, leg. P. Pavlović, Aug. 1909, one specimen (NHMBEO447).

##### Distribution and habitats in Serbia.

Known only from several localities in western Serbia (Fig. 15). Judging by the literature, found in areas rich in limestone.

##### Remarks.

The material of this species collected by Pavlović (see under the Material examined) needs revision. The sample of *V.illyrica* collected by Pavlović (NHMBEO449) is missing from the NHMBEO collection.

#### ﻿ Vitrea kiliasi

Taxon classificationAnimaliaStylommatophoraPristilomatidae

L. Pintér, 1972

108F3E4B-16FB-5638-835F-2B11D2DD2B55

Figs 5 , 15

Vitrea kiliasi — Karaman 2007: 141; Welter-Schultes 2012: 362.

##### Sites in Serbia known from the literature.

After Welter-Schultes (2012): near the city of Peć, Kosovo and Metohija.

**Figure 5.**
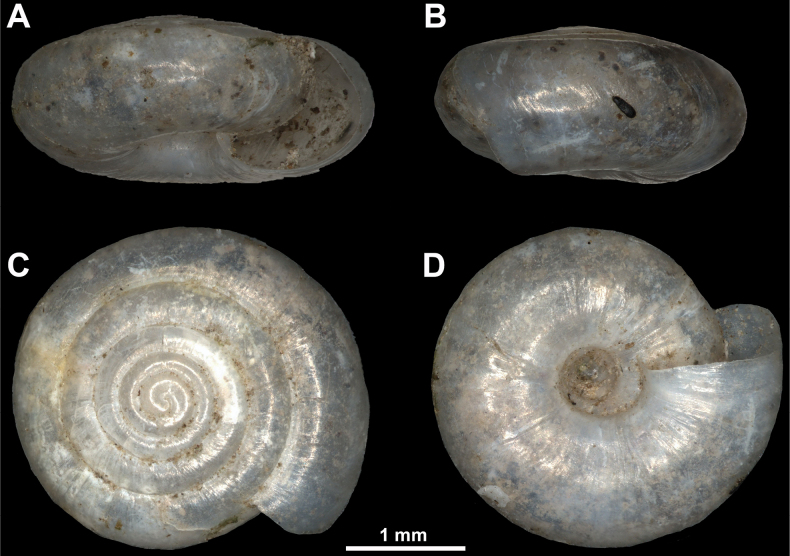
*Vitreakiliasi* L. Pintér, 1972 from Rugovska Klisura Gorge, Kosovo and Metohija (MIZ.MOL047276) **A** apertural view **B** lateral view **C** apical view **D** umbilical view (photo: Magdalena Kowalewska-Groszkowska).

##### Material examined.

**Serbia** • City of Peć, Rugovska Klisura Gorge, coll. W. Maassen, 12 Sep. 1987 (MIZ.MOL047276).

##### Description of specimens from Serbia.

Shell colourless, consisting of five regularly increasing, radially striated whorls. Last whorl ~ 1.5× wider than penultimate whorl. Periphery rounded, aperture elliptical. Umbilicus very wide, clearly showing all previous whorls.

##### Distribution and habitats in Serbia.

Known from a very limited geographical area in Kosovo and Metohija (Fig. 15). Habitat in Serbia unknown.

##### Remarks.

Welter-Schultes (2012) provided a photograph of this species from the surroundings of the city of Peć (Kosovo and Metohija). Otherwise, this species was not collected during our surveys.

#### ﻿ Vitrea kutschigi

Taxon classificationAnimaliaStylommatophoraPristilomatidae

(Walderdorff, 1864)

D9060563-C87E-5B5A-867C-695722824410

Figs 6 , 15

Vitrea kutschigi — Pintér 1972: 262; Karaman 2007: 141.

##### Sites in Serbia known from the literature.

After Pintér (1972): village of Bare, near the town of Sjenica.

**Figure 6.**
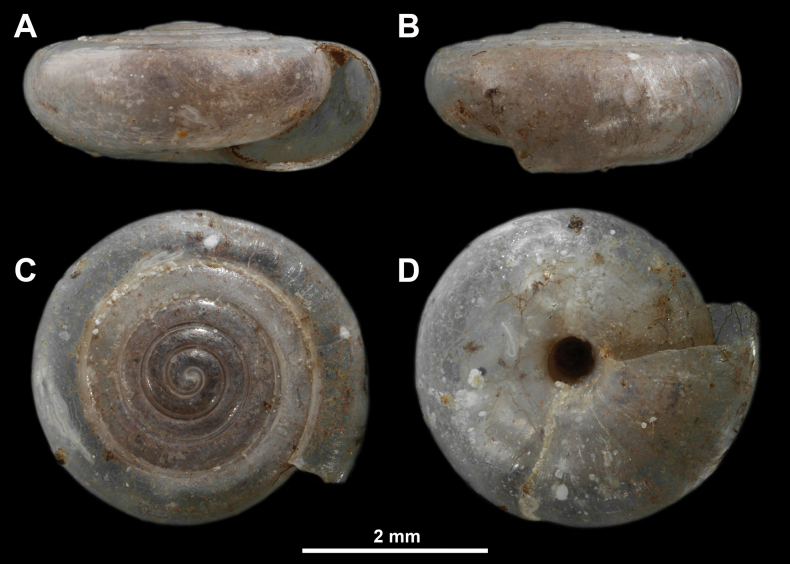
Neotype of *Vitreakutschigi* from Lokrum island, Croatia (SMF171014) **A** apertural view **B** lateral view **C** apical view **D** umbilical view.

##### Types examined.

Lokrum island, city of Dubrovnik, Croatia, neotype (SMF171014).

##### Other material examined.

None.

##### Distribution and habitats in Serbia.

This species is known only from limestone habitats in a limited part of western Serbia (Fig. 15).

##### Remarks.

A snail specimen from Mt. Tara (Drundebo) collected by Pavlović and deposited in the NHMBEO collection as *V.illyrica* (NHMBEO450) could actually refer to *V.kutschigi*, as its shell morphology differs (the shell is flatter, with more densely coiled whorls) from that of *V.illyrica*. The neotype of *V.kutschigi* was apparently designated by L. Pintér. The original material of Walderdorff (received by Parreyss) was lost, and the neotype was selected from the original material of Parreyss in the SMF collection (for details see Pintér 1972).

#### ﻿ Vitrea pygmaea

Taxon classificationAnimaliaStylommatophoraPristilomatidae

(O. Boettger, 1880)

3F4F7181-ADE4-51B9-8389-B0F70A8E58DE

Figs 7 , 15

##### Previous records from Serbia.

This species has not been previously reported from Serbia.

##### Material examined.

**Serbia** • Mt. Zlatibor, town of Čajetina, village of Gostilje, Gostilje waterfall, found among soil on limestone rocks, leg. V. Gojšina, 07 Aug. 2020, one specimen (43°39'24.83"N, 19°50'18.54"E).

**Figure 7.**
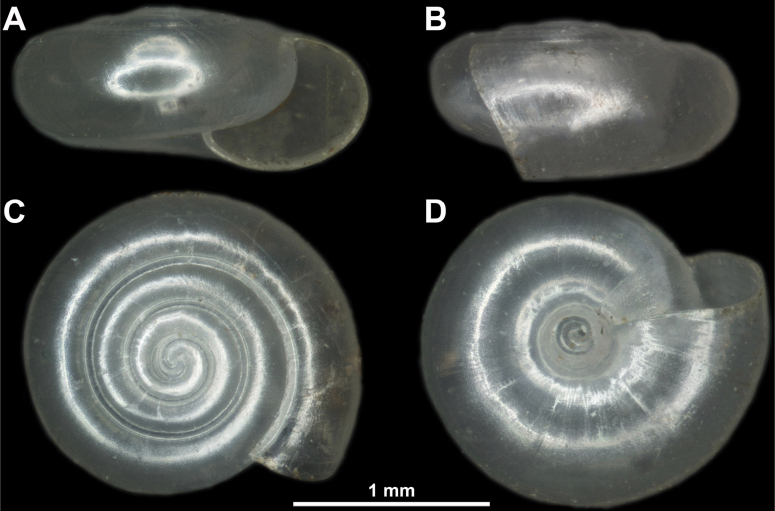
*Vitreapygmaea* from the vicinity of the Gostilje waterfall on Mt. Zlatibor **A** apertural view **B** lateral view **C** apical view **D** umbilical view.

##### Description of specimens from Serbia.

Shell very small (SW = 1.80 mm, SH = 0.82 mm), colourless and translucent. It consists of ~ 3.75 whorls separated by relatively deep suture. Aperture elliptical, periphery well rounded. Umbilicus broad, measuring ~ ¼ of SW and showing all previous whorls. Last whorl between 1.5 and 2.0× as wide as penultimate whorl.

##### Distribution and habitats in Serbia.

This species is only known from a single locality in western Serbia (Fig. 15), but is possibly more widespread. The small number of records to date is probably due to its tiny size and the lack of thorough sampling. It was found in soil samples from limestone rocks near the Gostilje waterfall.

##### Remarks.

The identification of this species is based on a single specimen and requires confirmation. In our specimen, the last whorl was ~ 1.5× wider than the penultimate whorl, which is slightly less than usually reported for this species (twice as wide or even wider). However, the SW, SH, number of whorls, and UW of this specimen match the values given in the description of this species (Pintér 1972).

#### ﻿ Vitrea sturanyi

Taxon classificationAnimaliaStylommatophoraPristilomatidae

(A. J. Wagner, 1907)

64EBD5F3-7CB0-58F2-B9BB-E4B4E41B0AC8

Figs 8 , 15

Crystallus sturanyi — Pavlović 1912: 27; Tomić 1959: 13.
Vitrea sturanyi — Jaeckel et al. 1957: 156; Karaman 2007: 141.

##### Sites in Serbia known from the literature.

After Pavlović (1912) and Tomić (1959): village of Gornje Košlje, near the town of Ljubovija; Drundebo, Mt. Tara; Mt. Kablar, near the city of Čačak.

**Figure 8.**
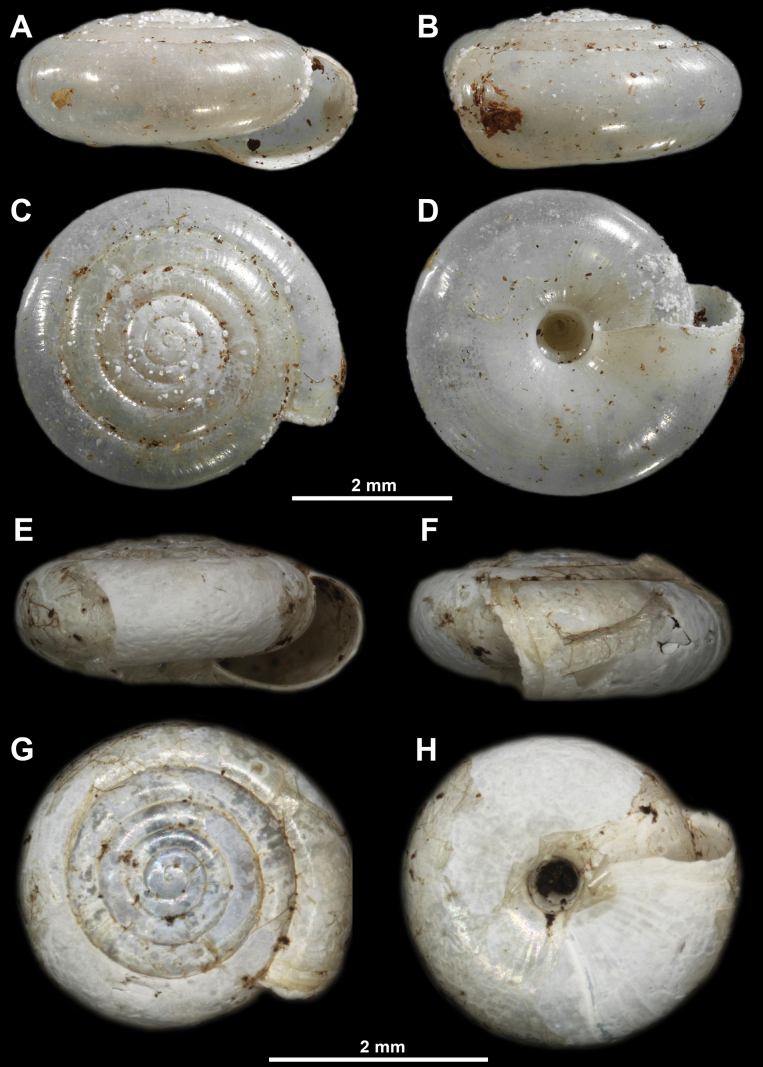
Paralectotype of *Vitreasturanyi* from Mt. Bjelašnica, Bosnia and Herzegovina (SMF171014) (**A–D**) and *V.sturanyi* from the village of Lukino Selo on Mt. Tara, Serbia (**E–H**) **A, E** apertural view **B, F** lateral view **C, G** apical view **D, H** umbilical view. The upper scale refers to photos **A–D**, while the lower scale refers to photos **E–H**.

##### Types examined.

Mt. Bjelašnica, Bosnia and Herzegovina, three paralectotypes (SMF171029).

##### Other material examined.

**Serbia** • Mt. Kablar, 23 Sep. 1908, one specimen (NHMBEO455); Mt. Tara, village of Lukino Selo, close to the Spajići Lake, next to a small brook connected to the Beli Rzav river, leg. D. Antić, M. Šević, D. Pavićević & I. Karaman, 06 Oct. 2023, two specimens (43°50'51.35"N, 19°23'48.68"E).

##### Description of specimens from Serbia.

Shell relatively large (SW = 3.25 mm, SH = 1.3 mm), consisting of ~ 5.5 densely coiled and regularly increasing whorls. Last whorl ~ 1.5× as wide as penultimate whorl. Periphery rounded, aperture relatively narrow. Umbilicus with perpendicular walls, UW measuring 1/6 of SW.

##### Distribution and habitats in Serbia.

In Serbia only known from a small number of sites rich in limestone in the west and southwest of the country (Fig. 15).

##### Remarks.

Only two weathered shells were available, so details of the surface sculpture could not be observed. The SW of the adult specimen (with ~ 5.5 whorls) was 3.25 mm, which is slightly less than indicated in the literature (Welter-Schultes 2012). The last whorl was significantly wider than the penultimate whorl, in contrast to the usual condition in which these two whorls have the same width. Other features perfectly match those of the paralectotype and those listed in the description of *V.sturanyi* by Welter-Schultes (2012) (Fig. 8). The sample of *V.sturanyi* collected by Pavlović (NHMBEO453) is missing from the NHMBEO collection.

#### ﻿ Vitrea subrimata

Taxon classificationAnimaliaStylommatophoraPristilomatidae

(Reinhardt, 1871)

91E14CD7-04A0-5E58-BD94-F9CD1E401D2A

Figs 9 , 16

Crystallus subrimatus — Pavlović 1912: 25–26.
Crystollus subrimatus [sic] — Tomić 1959: 12–13.
Hyalina subrimata — Möllendorff 1873: 131.
Vitrea submata [sic] — Jovanović 1993: 242.
Vitrea subrimata — Jaeckel et al. 1957: 156; Pintér 1972: 231; Jovanović 1985: 42; Sólymos et al. 2004: 152; Karaman 2012: 24.

##### Sites in Serbia known from the literature.

After Möllendorff (1873): Rača Monastery, Mt. Tara; after Pavlović (1912) and Tomić (1959): Mt. Avala, near the city of Belgrade; Jerinin Grad, near the town of Batočina; Mt. Belava, near the town of Bela Palanka; Mt. Beljanica; Mt. Vidlič, near the city of Pirot; Visoka Klisura Gorge, near the Veliki Rzav river; village of Gornje Košlje, near the town of Ljubovija; Mt. Golija, near the town of Ivanjica; Mt. Goč, near the town of Vrnjačka Banja; next to the Dubočica river, near the town of Raška; Derventa river canyon, Mt. Tara; Drundebo, Mt. Tara; Zečki Vrh peak, Mt. Čemernica; Mt. Javor; Jankova Klisura Gorge, village of Čučale, near the town of Blace; Radmanov Kamen, Mt. Kopaonik; Metođe, Mt. Kopaonik; Majića Krš, Mt. Kopaonik; Srebrnac, Mt. Kopaonik; Kadijina Stena, near Mt. Javor; Kamenova Kosa (?); Krstača, near the Rača Monastery, Mt. Tara; Marića Stena, near the town of Krupanj; village of Lepena, near the town of Knjaževac; Murtenica mountain massif, Mt. Zlatibor; Mt. Mučanj; Mt. Medvednik; near the Panjica river, village of Dobrače, near the town of Ivanjica; Proslop saddle, near the city of Valjevo; Pustinja Monastery, village of Poćuta, close to the city of Valjevo; Mt. Povlen; near the Prištavica river, Mt. Zlatibor; village of Rti, near the town of Lučani; Sićevo Gorge, near the city of Niš; Ulanac peak, Svrljiške Planine Mts.; Glogovački Vrh peak, Mt. Tupižnica; Tornička Bobija peak, Mt. Bobija, near the town of Ljubovija; village of Taor, near the city of Valjevo; surroundings of the city of Užice; village of Crnoljevica, near the town of Svrljig; after Pintér (1972): Mt. Avala, near the city of Belgrade; surroundings of the city of Užice; village of Bare, near the town of Sjenica; after Jovanović (1985): Mt. Avala, near the city of Belgrade; after Sólymos et al. (2004) and Karaman (2012): near the Veliki Potok stream, Mt. Fruška Gora.

**Figure 9.**
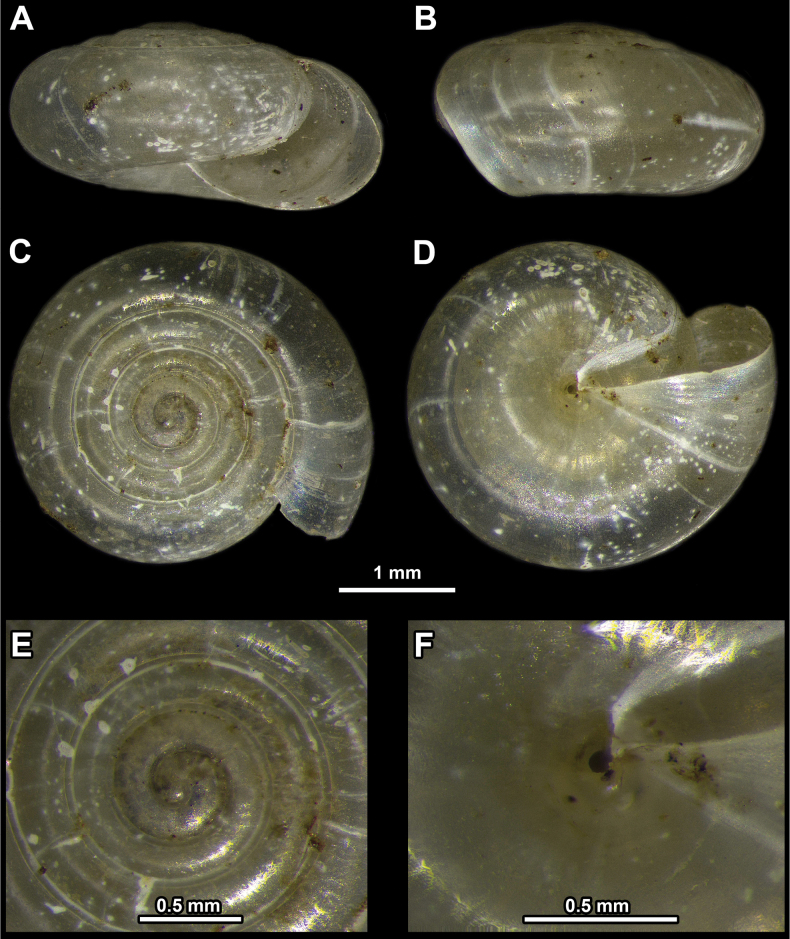
*Vitreasubrimata* from near the Bigar waterfall on Stara Planina Mts. **A** apertural view **B** lateral view **C** apical view **D** umbilical view **E** enlarged view of the protoconch **F** enlarged view of the umbilicus.

##### Material examined.

**Serbia** • Town of Knjaževac, village of Lepena, 08 Jun. 1907, one specimen (NHMBEO437); Mt. Tupižnica, Glogovački Vrh peak, leg. V. Petković, 1907, one specimen (NHMBEO435); Mt. Jadovnik, near the Studenac spring, leg. M. Vujić, 16 Sep. 2021, one specimen (43°18'31.64"N, 19°47'50.43"E); Mt. Jadovnik, Katunić peak, leg. V. Gojšina, N. Vesović & S. Ćurčić, 25 Jun. 2023, one specimen (43°16'27.62"N, 19°50'23.36"E); city of Bor, Mt. Stol, leg. V. Gojšina, 18 Jun. 2022, one specimen (44°10'17.40"N, 22°07'34.78"E); Mt. Kosmaj, village of Ralja, near a spring close to the Hotel “Babe”, leg. V. Gojšina, 16 Apr. 2022, two specimens (44°32'17.85"N, 20°30'58.05"E); Stara Planina Mts., near the Bigar waterfall, leg. V. Gojšina, 05 Aug. 2022, one specimen (43°21'16.13"N, 22°26'33.02"E); Stara Planina Mts., surroundings of the village of Oreovica, leg. M. Šćiban, 30 Apr. 2012, three specimens; city of Belgrade, Stepin Lug park-forest, among rocks, leg. V. Gojšina & M. Vujić, 04 Apr. 2022, four specimens (44°44'50.26"N, 20°32'02.99"E); town of Tutin, village of Đerekare, among limestone rocks, leg. V. Gojšina, 25 Oct. 2022, one specimen (42°59'23.98"N, 20°07'47.37"E); Jelašnica Gorge, near the city of Niš, on limestone rocks, leg. V. Gojšina, 28 May 2022, two specimens (43°16'45.82"N, 22°03'49.59"E); Đerdap National Park, village of Brnjica, leg. V. Gojšina, M. Vujić & N. Vesović, 05 May 2023, three specimens (44°39'23.44"N, 21°46'01.26"E); Đerdap National Park, village of Dobra, leg. V. Gojšina, M. Vujić & N. Vesović, 05 May 2023, two specimens (44°38'27.53"N, 21°54'29.38"E).

##### Description of specimens from Serbia.

SW ranging from 3 to 4 mm. Shell surface smooth. Shell transparent, consisting of 4–5 moderately densely coiled whorls separated by shallow suture. Periphery rounded. Last whorl slightly < 2× as wide as penultimate whorl. Umbilicus very narrow, but clearly open, slightly covered by reflected columellar margin. Previous whorls not visible through umbilicus.

##### Distribution and habitats in Serbia.

Together with *V.diaphana*, this is the most common *Vitrea* species in Serbia (Fig. 16). Most frequently found in areas rich in limestone.

#### ﻿ Vitrea virgo

Taxon classificationAnimaliaStylommatophoraPristilomatidae

Gojšina & Dedov sp. nov.

A30FDCEE-D11D-556A-813B-A10F8246BA21

https://zoobank.org/350CE916-5C91-4D50-9667-B03DCDB82FE8

**Figure 10.**
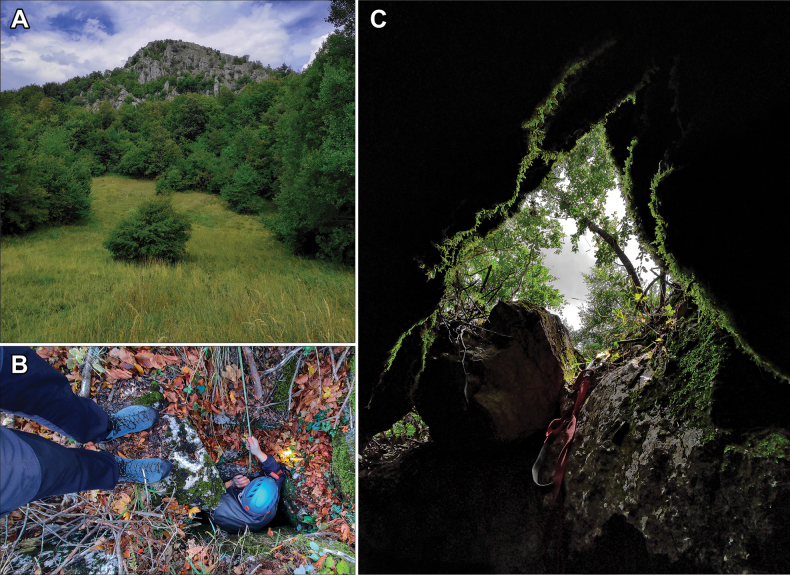
Type locality of *Vitreavirgo* sp. nov. **A** Oštra Čuka peak **B** entrance to the Jama pod Oštrom Čukom Pit, where the type specimens of *Vitreavirgo* sp. nov. were found **C** photo from inside the pit.

**Figure 11.**
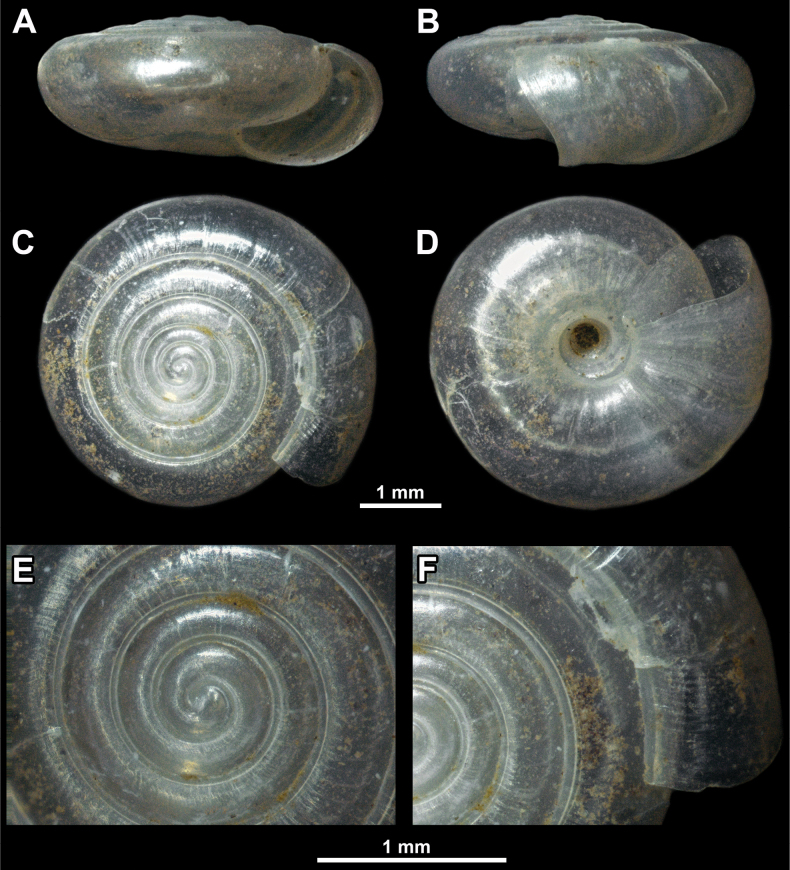
*Vitreavirgo* sp. nov. from Mt. Devica (holotype, NHMBEO312) **A** apertural view **B** lateral view **C** apical view **D** umbilical view **E** enlarged view of the protoconch **F** enlarged view of the last and penultimate whorl.

**Figure 12.**
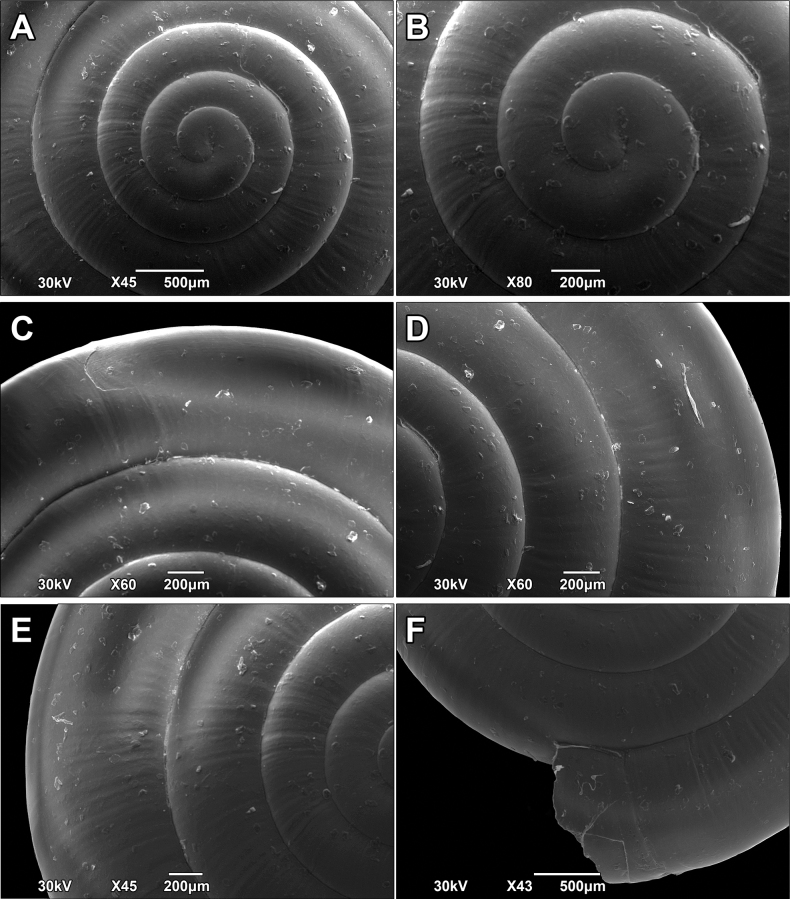
SEM images of the shell surface structure of *Vitreavirgo* sp. nov. **A, B** enlarged view of the protoconch **C–F** enlarged different parts of the last whorl.

**Figure 13.**
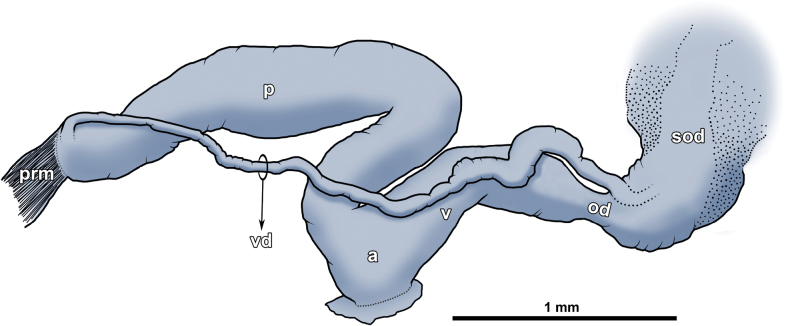
Genitalia of *Vitreavirgo* sp. nov. (paratype, IZOO-MG-014). **a** genital atrium **od** oviduct **p** penis **prm** penial retractor muscle **sod** spermoviduct **v** vagina **vd** vas deferens.

**Figure 14.**
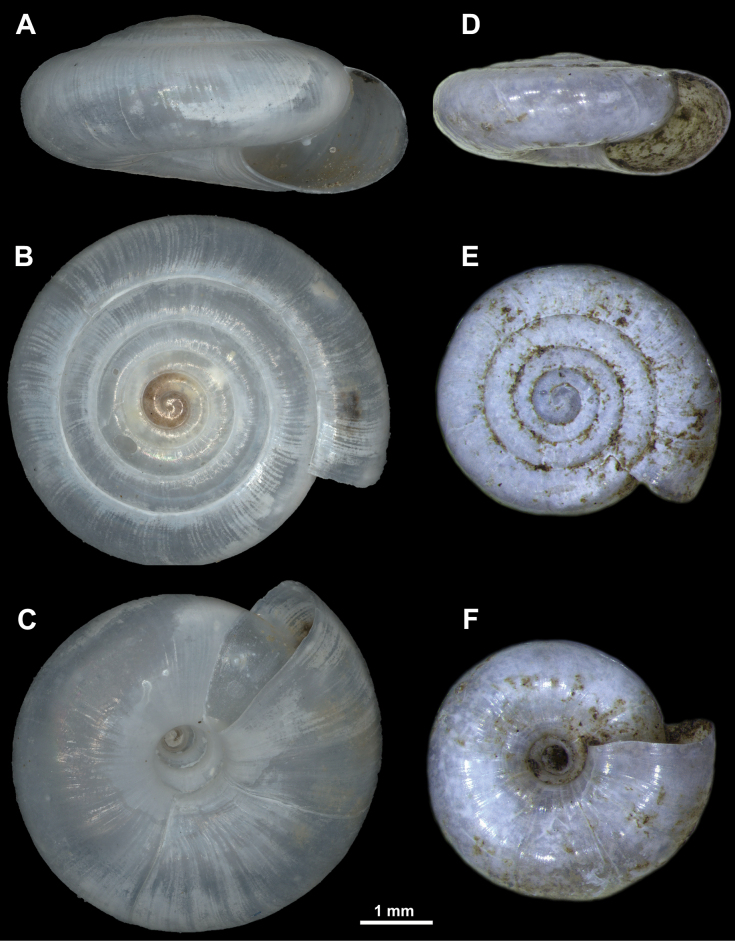
**A–C***Vitreasiveci* from Solunska Glava peak on Mt. Jakupica, North Macedonia (paratype, MIZ.MOL047322) (photo: Magdalena Kowalewska-Groszkowska) **D–F***V.virgo* sp. nov. from Mt. Devica (paratype, NHMBEO313) **A, D** apertural view **B, E** apical view **C, F** umbilical view.

**Figure 15.**
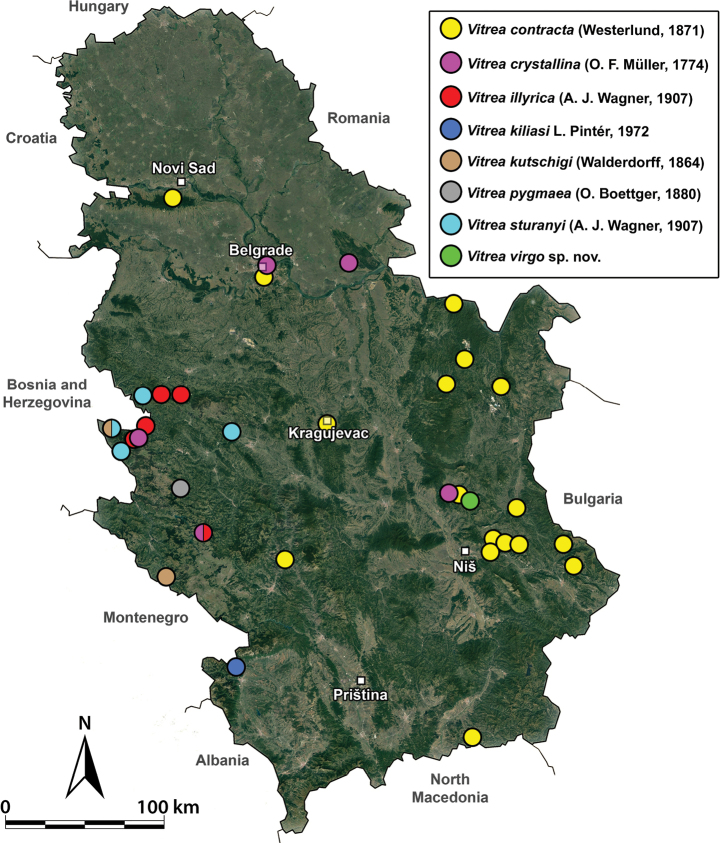
A distribution map of *Vitreacontracta*, *V.crystallina*, *V.illyrica*, *V.kiliasi*, *V.kutschigi*, *V.sturanyi*, and *V.virgo* sp. nov. in Serbia.

Figs 10 , 11 , 12 , 13 , 14D–F , 15

##### Type material.

***Holotype***: one dry-preserved shell (NHMBEO312), leg. V. Gojšina, N. Vesović & S. Ćurčić, 12 Aug. 2022. ***Paratypes***: 11 shells [codes: NHMBEO313 - one specimen (dry-preserved), IBER20469 - four specimens (ethanol-preserved), IZOO-MG-013 - two specimens (ethanol-preserved) and IZOO-MG-016 - four specimens (dry-preserved: one broken, one juvenile and two whole)] + genitalia in 70% ethanol (IZOO-MG-014).

##### Type locality.

**Serbia** • E Serbia, town of Sokobanja, Mt. Devica, Oštra Čuka peak, Jama pod Oštrom Čukom Pit, 1,033 m a.s.l. (43°35'38.48"N, 21°53'54.97"E).

##### Diagnosis.

The new species differs clearly from most of the congeners by the large size of the shell (SW usually > 4 mm in adults), densely coiled, radially striated whorls, and a wide umbilicus. At first glance, this combination of characteristics places this species close to the genera *Lindbergia* and *Spinophallus*, from which it differs in its genital anatomy. There are several species that have similar number of whorls and UW: *V.siveci*, *V.kutschigi*, *V.neglecta* Damjanov & L. Pintér, 1969, *V.bulgarica* Damjanov & L. Pintér, 1969, *V.illyrica*, and *V.kiliasi*. From the similar *V.siveci*, described from North Macedonia and present in Greece, the new species differs by the flatter shell, narrower last whorl and aperture, and less regularly rounded periphery. The umbilicus is larger and usually more distinctly funnel-shaped in the new species than in *V.siveci*, whose shell is larger (both in SW and SH) than in the new species. Namely, the SW of the largest specimen of *V.siveci* is 5.3 mm (Riedel and Velkovrh 1976), which is almost 0.7 mm more than in the largest specimen of *V.virgo* Gojšina & Dedov, sp. nov. in our sample (SW 4.68 mm). In addition, the surface sculpture is much more pronounced in *V.siveci* than in the new species. The western Balkan species *V.kutschigi* differs from the new species by its flatter shell and narrower aperture, which makes it similar to the freshwater planorbid species *Bathyomphaluscontortus* (Linnaeus, 1758), as observed by Welter-Schultes (2012). In addition, *V.kutschigi* is larger than *V.virgo* Gojšina & Dedov, sp. nov. and has a less pronounced funnel-shaped umbilicus. In *V.sturanyi*, another similar western Balkan species, the last whorl is as broad as the penultimate whorl (see under the Remarks section for *V.sturanyi*), and the umbilicus has almost perpendicular walls, which do not expand as much as in the new species. Finally, the shell of the new species is flatter and less rounded than in *V.sturanyi*. The shell of *V.illyrica* is less flat on both the upper and lower sides, the aperture is less narrow, the last whorl is less narrow and the whorls are less densely coiled than in the new species. Two Bulgarian species, *V.bulgarica* and *V.neglecta* (considered conspecific by Irikov 2001 and Welter-Schultes 2012, but treated as separate by Georgiev and Dedov 2014) are both smaller (SW usually ≤ 3.2–3.4 mm and SH ≤ 1.6 mm) and more conical, with usually less wide perspective umbilicus than in the new species. Spiral striation is not observed in these two species, but is present (albeit very weak and localised) in *V.virgo* Gojšina & Dedov, sp. nov. These two species also differ from the new species in their genital anatomy. According to Irikov (2001) and Georgiev (2016), *V.bulgarica* and *V.neglecta* have a penis with a strong bulge (swelling) distally and a well-developed perivaginal gland. In *V.virgo* Gojšina & Dedov, sp. nov., the penis is with no such strong swellings, thus almost equally broad throughout its entire length and no perivaginal gland is observed. In a specimen of *V.neglecta* from Greece, Georgiev (2016) noted that its mantle is speckled, with black-greyish pigmentation, in contrast to the new species, whose mantle is completely devoid of pigmentation. Differences in the appearance of the reproductive system are also observed when comparing the new species with other geographically close Serbian congeners (*V.contracta*, *V.crystallina*, *V.diaphana*, and *V.subrimata*). In contrast to them, the new species lacks both the seminal receptacle and the perivaginal gland. Compared to *Vitreaulrichi* Georgiev & Dedov, 2014, the new species has less whorls in the same SW (the shell in *V.ulrichi* is more densely coiled than in the new species) (in *V.ulrichi*, SW ~ 4.6 mm = 6.25 whorls *vs.* in *V.virgo* Gojšina & Dedov, sp. nov., SW ~ 4.6 mm = 5.5 whorls). Finally, the shells of *V.ulrichi* are more coarsely radially striated compared to those of *V.virgo* Gojšina & Dedov, sp. nov.

##### Description.

***Shell*** — Flat, translucent, consisting of 4.5–5.5 regularly increasing, densely coiled whorls separated by moderately deep suture. Protoconch smooth (Fig. 12A, B), consisting of ~ 1.25–1.5 whorls. Boundary between protoconch and teleoconch slightly visible only by scanning electron microscopy (SEM) and even then not clear. Teleoconch almost smooth, but with several very fine, irregular radial growth lines. Spiral striation very weak, present only on some parts of periphery, composed of innumerable spiral lines that are very difficult to observe (Fig. 12C). Lower side of shell almost flat. Last whorl on average 1.5× (sometimes ≤ 1.7×) as wide as penultimate whorl. Peristome sharp, almost straight when observed from apical view. Aperture elliptical and relatively narrow. Umbilicus wide, measuring 1/5–1/6 of SW and showing almost all whorls inside. Surface sculpture much less distinct (almost invisible) on umbilical side when compared to apical side.

##### Reproductive system.

Genitalia typical for *Vitrea*. Penis moderately long, almost of equal width along entire length, very slightly widening only medially. Penial retractor muscle inserted at apical part of penis, where vas deferens joins too. Latter structure long and very thin, but thickened near female part of genitalia. Epiphallus and seminal receptacle absent. Genital atrium indistinct. Vagina almost as wide as penis. Perivaginal gland could not be observed, probably absent (Fig. 13).

##### Measurements

**(in mm, *n* = 7)**: SW = 3.61–4.68; SH = 1.54–2.10; AW = 1.57–1.87; AH = 1.22–1.50; UW = 0.61–0.86.

##### Etymology.

The new species is named after Mt. Devica, where the type locality (Jama pod Oštrom Čukom Pit) is situated. The name of the mountain means “a virgin” (Lat. *virgo*) in Serbian. The specific epithet is to be used as a noun in apposition.

##### Habitat.

The new species is found in a shallow, natural pit (a small underground cavern between boulders) several meters deep in a limestone habitat. Live animals crawled on and under numerous wet rocks deeper in the pit. They were only found in the darker parts of the pit. The new species was found together with two other gastropods, *Morlinaglabra* (Rossmässler, 1835) and *Limaxcinereoniger* Wolf, 1803. It was not found outside the pit, although it may also occur in the immediate vicinity.

##### Distribution.

This species is only known from the type locality (Figs 10, 15).

##### Remarks.

The radial striation of the shell is irregular and quite variable in the new species. In some places, the shells appear to be almost completely smooth or, on the contrary, show strong radial lines. *Vitreavirgo* Gojšina & Dedov, sp. nov. is one of the largest representatives of the genus *Vitrea* in Serbia. Based on this fact, we had suspected that it might even belong to several other genera with typically larger shells [for the dimensions of the species see Welter-Schultes (2012)], such as *Lindbergia* and *Spinophallus*. After its dissection, however, we found no seminal receptacle, which is typically large and well developed in the other two genera, but absent or reduced in *Vitrea* (Schileyko 2003). More importantly, we found no epiphallus, which justifies the placement of the new species in the genus *Vitrea*. On the vas deferens we found a “seminal receptacle-like” structure whose function or origin is unknown. We are not sure what this structure represents, and it is probably an artefact, as it was not observed in any other dissected specimen.

**Figure 16.**
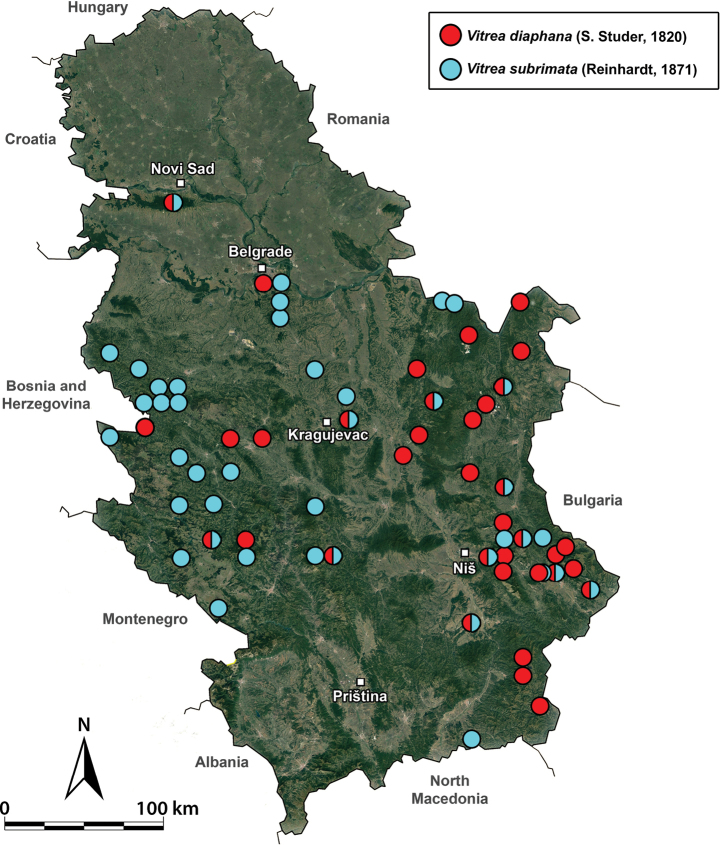
A distribution map of *Vitreadiaphana* and *V.subrimata* in Serbia.

### ﻿Identification key to the species of the genus *Vitrea* from Serbia

**Table:** 

1	Umbilicus narrow or wide, never closed	**2**
–	Umbilicus closed	***V.diaphana***
2	Umbilicus moderately to very wide	**3**
–	Umbilicus very narrow	***V.subrimata***
3	Last whorl wider than penultimate whorl	**4**
–	Last whorl of the same width as penultimate whorl	***V.sturanyi***
4	Shell smaller, width ≤ 4 mm in adults	**5**
–	Shell larger, width > 4 mm in adults	**8**
5	Umbilicus very wide	**6**
–	Umbilicus not very wide	**7**
6	Shell ≤ 2 mm wide, last whorl mostly twice as wide as penultimate whorl	***V.pygmaea***
–	Shell 3–3.3 mm wide, last whorl 1.5× as wide as penultimate whorl	***V.kiliasi***
7	Whorls not densely coiled, umbilicus moderately wide, SW usually between 3 and 4 mm	***V.crystallina***
–	Whorls relatively densely coiled, SW usually ~ 2 mm	***V.contracta***
8	Shell flat, last whorl and aperture relatively narrow, whorls densely coiled	**9**
–	Shell convex, last whorl and aperture wide, whorls not very densely coiled	***V.illyrica***
9	Shell very flat, aperture very narrow, umbilicus with perpendicular walls, shell surface sculpture not very prominent, last whorl sometimes appears slightly shouldered	***V.kutschigi***
–	Shell moderately flat, aperture moderately narrow, umbilicus wide, especially at last whorl, shell surface sculpture prominent, last whorl regularly rounded	***V.virgo* sp. nov.**

## ﻿Discussion

This study increases the total number of *Vitrea* species in Serbia to 10. The specific diversity of this genus in neighbouring countries varies between five and 13. Five species are known from Hungary (Pintér and Suara 2004), seven from Albania (Fehér and Erőss 2009), nine from Montenegro (Karaman 2014), 10 from Bosnia and Herzegovina (Karaman 2006) and Romania (Bank and Neubert 2017), 11 from Bulgaria (Georgiev and Dedov 2014), while 13 species are known from North Macedonia (Maassen 1998; Stankovic et al. 2006; Dedov 2021) and Croatia (Štamol 2010). None of the known Serbian species is endemic to this country, with the exception of the newly described species. However, several species reported here are western Balkan endemics (*V.illyrica*, *V.kiliasi*, *V.kutschigi*, and *V.sturanyi*) (Pintér 1972; Welter-Schultes 2012). The species newly reported for the Serbian fauna, *V.pygmaea*, was found in a region within the range of the species, which means that the lack of previous records is probably due to a lack of research. Eastern Serbia can be considered the best-studied part of the country in terms of snail fauna, including the representatives of the genus *Vitrea* (Pavlović 1912; Jovanović 1993, 1996). Pavlović (1912) provided data for many gastropod species from this region with a dense network of sampling sites. This is also the region where most endemic Serbian gastropod taxa are found (Karaman 1999; Subai 2011). Apart from numerous samplings in the past, the only *Vitrea* representatives found in eastern Serbia are the most widespread species (*V.contracta*, *V.diaphana*, and *V.subrimata*). The abovementioned endemics of the western Balkans, on the other hand, are more common in western Serbia (Pavlović 1912).

The perivaginal gland is an organ that is frequently found in gastrodontoid and zonitoid snails (Schileyko 2003). This organ can vary in size and shape in different taxa and can also be positioned differently (Rodrigues et al. 2002), but is usually located near the vagina and the distal part of the free oviduct (near where the bursa normally attaches). The function of this organ is related to egg-shell production and lubrication of the distal female genitalia, and its secretions are composed of proteins, mucopolysaccharides, and calcium (Rodrigues et al. 2002). Although this is not the rule, the perivaginal gland can sometimes be completely absent (e.g., Riedel 1960; Slapcinsky 2018). We did not observe a perivaginal gland in the dissected specimens of *V.virgo* Gojšina & Dedov, sp. nov.

The distribution of even common species (e.g., *V.crystallina*) in Serbia is still poorly known, as there are few records in the country due to the following two facts: i) all *Vitrea* species are relatively small and usually difficult to find *in situ*, which is why soil sampling is recommended; and ii) knowledge about terrestrial snails in Serbia is still very poor due to the lack of experts and short research tradition. Further sampling and research are needed to fully understand the distribution of species, especially those that occur in specialised habitats and are known only from a few localities. The species narrowly distributed in Serbia (*V.illyrica*, *V.kiliasi*, *V.kutschigi*, and *V.sturanyi*) may be threatened by habitat changes, especially because they are restricted to limestone areas that are frequently quarried (for some examples see Schilthuizen et al. 2005). We are not yet in a position to assess the actual threat to these species in Serbia, as their distribution in this country is largely unknown. The type locality of the newly described species is not yet under high anthropogenic pressure, as its surroundings are currently not threatened by quarry work. However, there is a potential threat in the form of habitat changes (waste dumping, deforestation, and urbanisation), as Mt. Devica could become a tourist attraction in Serbia. The actual distribution of the newly described species and its habitat preferences need to be further investigated in order to adequately protect the species and its habitat, should this become necessary.

Although considerable efforts were made to sample terrestrial gastropods at several other sites on Mt. Devica and its surroundings, the new species was only found at its type locality. It is possible that this species is subterranean, as no specimens were found outside the pit and the specimens we collected had no mantle pigmentation, which is consistent with other subterranean taxa. *Vitreavirgo* Gojšina & Dedov, sp. nov., like many other relatives (Welter-Schultes 2012), could be a rare species with a restricted geographic distribution, but further sampling and study is needed to verify its narrow range and specific microhabitat requirements.

## Supplementary Material

XML Treatment for Vitrea

XML Treatment for Vitrea contracta

XML Treatment for Vitrea crystallina

XML Treatment for Vitrea diaphana

XML Treatment for Vitrea illyrica

XML Treatment for Vitrea kiliasi

XML Treatment for Vitrea kutschigi

XML Treatment for Vitrea pygmaea

XML Treatment for Vitrea sturanyi

XML Treatment for Vitrea subrimata

XML Treatment for Vitrea virgo

## ﻿Additional information

### Author ORCIDs

Vukašin Gojšina https://orcid.org/0000-0002-0413-9304

Nikola Vesović https://orcid.org/0000-0001-6256-7975

Srećko Ćurčić https://orcid.org/0000-0001-7303-7857

Tamara Karan-Žnidaršič https://orcid.org/0000-0003-3821-578X

Ivaylo Dedov https://orcid.org/0000-0003-4445-359X

### Data availability

All of the data that support the findings of this study are available in the main text.
